# Fatal Cowpox Virus Infection in Human Fetus, France, 2017

**DOI:** 10.3201/eid2710.204818

**Published:** 2021-10

**Authors:** Audrey Ferrier, Gaelle Frenois-Veyrat, Evelyne Schvoerer, Sandrine Henard, Fanny Jarjaval, Isabelle Drouet, Hawa Timera, Laetitia Boutin, Estelle Mosca, Christophe Peyrefitte, Olivier Ferraris

**Affiliations:** Institut de Recherche Biomédicale des Armées, Brétigny-sur-Orge, France (A. Ferrier, G. Frenois-Veyrat, F. Jarjaval, I. Drouet, H. Timera, L. Boutin, E. Mosca, C. Peyrefitte, O. Ferraris);; Centre Hospitalier Régional Universitaire, Nancy, France (E. Schvoerer, S. Henard)

**Keywords:** cowpox virus, fetal mortality, infection, viruses, France, orthopoxvirus

## Abstract

Cowpox virus (CPXV) has an animal reservoir and is typically transmitted to humans by contact with infected animals. In 2017, CPXV infection of a pregnant woman in France led to the death of her fetus. Fetal death after maternal orthopoxvirus (smallpox) vaccination has been reported; however, this patient had not been vaccinated. Investigation of the patient’s domestic animals failed to demonstrate prevalence of CPXV infection among them. The patient’s diagnosis was confirmed by identifying CPXV DNA in all fetal and maternal biopsy samples and infectious CPXV in biopsy but not plasma samples. This case of fetal death highlights the risk for complications of orthopoxvirus infection during pregnancy. Among orthopoxviruses, fetal infection has been reported for variola virus and vaccinia virus; our findings suggest that CPXV poses the same threats for infection complications as vaccinia virus.

Cowpox virus (CPXV) is a member of the genus *Orthopoxvirus* in the family *Poxviridae*. CPXV is assumed to be the causative agent of cowpox, mainly associated with lesions on the udders of dairy cows and the hands of dairy workers. This zoonotic disease has a broad range of hosts (*1*), so spillover infections to accidental hosts (e.g., rats, cats, cattle, horses, llamas, zoo animals, and humans) are reported regularly; case numbers in Europe are increasing (*2*).

CPXV and all other orthopoxviruses that can infect humans (with the exception of variola virus) have an animal reservoir and are transmitted to humans by contact with infected animals. Wild rodents (voles) are considered the reservoir host species (*3*), but zoonotic transmissions of CPXV have been mainly caused by direct contact with infected pet rats (*4*–*7*), cats (*8*,*9*), or zoo animals (*10*,*11*).

Among orthopoxviruses, infection of the human fetus has been reported for variola virus and vaccinia virus (*12*–*15*). Congenital vaccinia is a rare complication of vaccination; <40 cases have been described in the literature (*16*), such as serious consequences to the fetus of vaccinated women, including death or premature birth (*16*,*17*). We describe a fatal case of CPXV infection in a human fetus.

## Materials and Methods

### The Case

**Figure 1:**
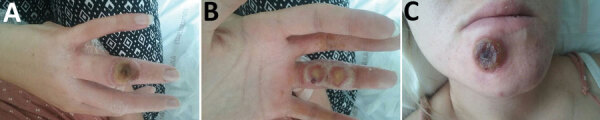
Cowpox virus infection of a 22-year-old pregnant woman with atopic dermatitis, France, July 13, 2017. A) Cutaneous lesion on the dorsal surface of finger on right hand. B) Cutaneous lesion on the palmar surface of finger on right hand. C) Cutaneous lesion on the chin.

On July 13, 2017, a 22-year-old pregnant woman (11 weeks of gestation, according to her last menstrual period) was admitted to Brabois University Hospital in Nancy, France. The patient arrived with multiple lesions on her right hand, 1 on the dorsal surface of a finger, and 2 on the palmar surface of the same finger (Figure 1, panels A, B). She also had a lesion on her chin (Figure 1, panel C), fever, ipsilateral axillary lymphadenopathy, and preexisting atopic dermatitis on her hands. The first lesion, on the dorsal surface of her right hand, seemed to have appeared on June 30, followed by the palmar lesions ≈3 days later. The patient stated that the lesion on her chin appeared later as a result of autoinoculation.

Since June 30, the patient had experienced 2 episodes of fever (38.5°C), on June 30 and July 12; she received antimicrobial therapy while hospitalized (July 13) to avoid superinfection. On July 18, the diagnosis of an orthopoxvirus-positive, smallpox virus–negative infection was confirmed by PCR at the hospital, and the National Reference Center–Expert Laboratory for Orthopoxvirus (Brétigny-sur-Orge, France) was contacted. DNA from a finger biopsy sample taken on July 18 was analyzed, as were cutaneous biopsy and plasma samples from July 19. The orthopoxvirus-positive, smallpox virus–negative diagnosis was confirmed by identification of CPXV DNA in all samples. Infectious CPXV was detected in biopsy samples only, not in plasma. Fetal death was declared on July 20, after echography examination.

**Figure 2:**
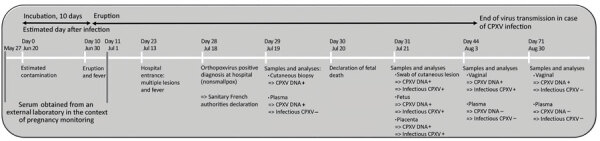
Chronology of CPXV infection of a 22-year-old pregnant woman, France, 2017, showing links between date of samples, detection of DNA or infectious CPXV, and course of the disease. Days after infection indicate the estimated day of infection based on the literature. CPXV, cowpox virus.

We identified CPXV DNA and infectious virus in fetal, placental, and cutaneous samples collected on July 21. On August 3, infectious CPXV was still detected in vaginal samples, whereas neither DNA nor infectious virus of CPXV was detected in the plasma. The last samples, collected on August 30, showed no infectious virus, only CPXV DNA, in vaginal samples; plasma was free of infectious virus and CPXV DNA (Table; Figure 2).

**Table:** Quantification of genomic or infectious CPXV in samples and CPXV-specific IgG detection in serum of mother and fetus, France, 2017*

Samples	Jul 19, 29 dpi			Jul 21, 31 dpi				Aug 3, 44 dpi			Aug 30, 71 dpi	
	Cutaneous biopsy	Plasma		Cutaneous biopsy	Fetus	Placenta		Vaginal swab	Plasma		Vaginal swab	Plasma
DNA, copies/μL	2.4×10^4^	26.6		8.6 × 10^6^	1.6 × 10^6^	1.4 × 10^6^		2.0 × 10^4^	–		27	–
Infectious virus, TCID_50_/mL	NT	–		1.86 × 10^7^	2.32 × 10^7^	9.74 × 10^7^		1.95 × 10^8^	–		–	–
CPXV-specific IgG	NA	++		NA	NA	NA		NA	++		NA	+++

*CPXV DNA was quantified by quantitative PCR. Infectious virus was titered on Vero cells. CPXV-specific IgG was detected by ELISA. The patient had domestic rabbits, cats and dogs at home. Given the probability of contamination of the animals, PCR was performed on claws and plasmas samples from the animals; all results were negative. CPXV, cowpox virus; dpi, days postinfection; NA, not applicable; NT, not tested; TCID_50_, 50% tissue culture infectious dose; ++, medium level; +++, high level.

Because CPXV incubation time is 8–12 days (*5*), we determined the incubation time for this case to be a mean of 10 days. Beginning with the appearance of signs and symptoms on June 30, we estimated the day of infection to be June 20, which corresponded to the ninth week of pregnancy, 10 days before symptom onset.

The patient had preexisting atopic dermatitis on her hands. She lived near a farm and had 2 rabbits as well as 2 dogs and 3 cats that were free to roam outside the house. The patient declared that she had neither touched her animals nor cleaned their litter since the beginning of her pregnancy.

### Virus Detection, Isolation, and Production

For virus detection, we extracted DNA from samples collected from pustular areas, blood, fetus, placenta, plasma, serum, and vaginal swabs in viral transport medium by using the QIAamp DNA Blood Mini Kit (QIAGEN, https://www.qiagen.com). We used 2 real-time quantitative PCR (qPCR) assays. The first assay detected orthopoxvirus on the basis of the A27L gene (*18*), by using the following primers: GF-5′-GCCAGAGATATCATAGCCGCTC, GR-5′-CAACGACTAACTAATTTGGAAAAAAAGAT, 14KD POX FAM/TAMRA-5′-TTTTCCAACCTAAATAGAACTTCATCGTTGCGTT and 14KD VAR FAM/TAMRA-5′-TTTTCCAACCTAAATAGAACGTCATCATTGCGTT. The second assay detected CPXV on the basis of the D8L and D11L genes by using the following primers: F-5′-GGTAGGTTCATGTTGGAAAATATC, R-5′-AAGATGTTATTAGTGGTATTAGAGAGAAAT, FAM/TAMRA-5-AAGTCATCTACTACATAGACCATGATCAACCAA (D8L gene) and F-5′-AAAACTCTCCACTTTCCATCTTCT; and R-5′-GCATTCAGATACGGATACTGATTC and FAM/TAMRA-5′-CCACAATCAGGATCTGTAAAGCGAGC (D11L gene). We conducted qPCR by using the iTaq Universal Probes Supermix (Bio-Rad, https://www.bio-rad.com).

To isolate competent virus, we used Vero cells maintained in Gibco Dulbecco’s modified Eagle medium GlutaMAX (DMEM-GlutaMAX) supplemented with 10% inactivated fetal bovine serum (FBSi; Thermo Fisher Scientific, https://www.thermofisher.com.) We inoculated 24-well plates of Vero cells with plasma and with fetal, placental, and vaginal swab samples, then placed the fetal, placental, and biopsy samples (from the mother’s hands) in phosphate-buffered saline (PBS) and crushed for 4 min at 30 Hz (TissueLyser; QIAGEN). We diluted supernatant of centrifuged samples (5 min at 1,500 × *g*) from 10^–2^ to 10^–6^ in DMEM-GlutaMAX, 0.4% FBSi, and 0.5 mg/mL of streptomycin and penicillin (Gibco; Thermo Fisher Scientific). We inoculated cells in duplicate with 450 μL of each sample and incubated them for 1 h at 37°C and 5% CO_2_. After incubation, we added 1.5 mL of DMEM-GlutaMAX, 0.4% FBSi, and 0.5 mg/mL of streptomycin and penicillin. After 8 days, we examined the cells for any cytopathic effects. For positive samples, we recovered the supernatants and stored them for amplification.

For virus amplification, we used Vero cells maintained in DMEM-GlutaMAX supplemented with 5% FBSi. After the cells reached 80% confluence, we removed the medium and inoculated the monolayer with 1 mL of viral suspension, which consisted of supernatant diluted to 1/10 in DMEM-GlutaMAX, 0.4% FBSi, and 0.5 mg/mL of streptomycin and penicillin. We incubated flasks at 37°C and 5% CO_2_ for 1 h for adsorption; then we removed the viral suspension and added 5 mL of DMEM-GlutaMAX, 2% FBSi, and 0.5 mg/mL streptomycin and penicillin. We incubated cells at 37°C and 5% CO_2_ for 3 d; on the third day, we freeze-thawed the flasks 3 times, then transferred the contents to 15-mL Falcon tubes and centrifuged at 1,200 × *g* for 10 min to pellet the cell debris. We removed the supernatant and aliquoted it for electronic microscopy and phylogenetic studies. We determined virus titers (expressed in terms of 50% tissue culture infectious dose [TCID_50_] per milliliter) by plaque assay in 96-well plates on Vero cells according to the Reed-Muench method (*19*).

### Transmission Electron Microscopy

For microscopy, we used BHK-21 cells maintained in Glasgow’s minimum essential medium, 10% FBSi, and 0.5 mg/mL streptomycin and penicillin. To infect the cells, we used virus isolated from fetal and placental samples as well as vaginal swab samples. After cells reached 80% confluence, we inoculated them with virus at a multiplicity of infection of 0.5. Two days after infection, we fixed cells with 2.5% (vol/vol) glutaraldehyde in sodium cacodylate buffer (0.1 M; pH 7.4; 1 mM CaCl_2_, 1 mM MgCl_2_, and 2% sucrose) for 1 h at 4°C. After washing samples with a mixture of saccharose (0.2 M) and sodium cacodylate (0.1 M), we postfixed them with 1% (vol/vol) osmium tetraoxide in cacodylate buffer for 1 h at room temperature. Then, we stained them with 2% (vol/vol) uranyl acetate for 1 h at 4°C and gradually dehydrated them with increasing ethanol concentrations. We embedded samples in Epon LX112 resin (Ladd Research Industries, https://www.laddresearch.com) in embedding capsules and polymerized for 24 h at 60°C. We then cut ultrathin 80-nm sections with an UC6 ultramicrotome (Leica, https://www.leica-microsystems.com), placed them onto 300-mesh copper grids, stained sections with 2% uranyl acetate and lead citrate, and examined them under a Philips CM10 TEM microscope (https://www.philips.nl) operating at 100 kV and equipped with a Denka LaB6 cathode (https://www.denka.co.jp) and a CCD Erlanghsenn 1000 Gatan camera (https://www.gatan.com). We applied no filtering procedures to the images.

### A56R Genome Sequencing

With regard to the ongoing case, we investigated 2 strains from our collection (CPXV-54-1716F1-France from the placenta specimen and CPXV-54-1716E1-France from the fetus specimen) and compared them with 7 CPXV strains from our strain collections (Cepad 332, Cepad 333, Cepad 336, Cepad 335, CPXV-85-1407-France, CPXV-35-1611-France, and CPXV-54-1405-France). We freeze-thawed infected cell cultures 3 times and extracted genomic DNA by using the QIAamp DNA Blood Mini Kit (QIAGEN). For molecular characterization, we amplified fragments containing the full A56R gene by PCR in a reaction volume of 50 μL containing 500 nM forward primer, 500 nM reverse primer, Q5 High Fidelity 2X Master Mix (New England BioLabs, https://www.neb.com) at 1× concentration, and 5 μL template DNA. The cycling conditions for both genes corresponded to initial denaturation at 98°C for 30 s; followed by 30 cycles at 98°C for 10 s, 55°C for 20 s, and 72°C for 45 s; and by final extension at 72°C for 2 min. We purified the amplified product of the A56R gene by using QIAquick PCR Purification Kit (QIAGEN), and sequencing was performed by Eurofins at Cochin Institut (Paris, France).

We assembled and edited the sequences by using Clone Manager 7 (Sci Ed Software, https://www.scied.com) and aligned the full-length A56R gene sequences by using BioEdit software version 7.0.9 (https://bioedit.software.com). For phylogenetic reconstructions of the A56R tree, we aligned sequences with MUSCLE by using the aligned codon option of MEGA 6 (https://www.megasoftware.net). We inferred the evolutionary history by using the neighbor-joining method of MEGA 6.

### IgG ELISA Data Processing and Normalization

For the IgG ELISA, we coated MaxiSorp microtiter plates (Dutscher, https://www.dutscher.com) with 4 μg/mL of purified vaccinia Copenhagen virus or noninfected cell lysate in carbonate buffer and incubated them overnight at 4°C. The plates were then blocked for 1 h at room temperature with Blocking Reagent (Roche France, https://www.roche.fr), followed by washing 3 times with PBS Tween (Fisher Scientific, https://fishersci.fr). We then added 2-fold serial dilutions in Antibody Diluent (HAMA Blocker) for ELISA (abcam, https://www.abcam.com), from 1:100 of orthopoxvirus-negative, orthopoxvirus-positive serum samples (human vaccinia Ig reference no. EDQM Y0000502), followed by patient serum samples and incubation for 2 h at 37°C. We washed the plates and added chicken anti-human IgG heavy and light chain horseradish peroxidase conjugate (abcam) at a 1:5,000 dilution (Antibody Diluent, abcam) for 1 h at 37°C. We then washed the plates again, added tetramethylbenzidine 1-component substrate (Sigma Aldrich, https://www.sigmaaldrich.com), and allowed development to colorimetric result to proceed for 15 min. We stopped the plate reactions by adding stop solution (H_3_PO_4_ 1M), and we read the optical density at 450 nm on a spectrophotometer (Tecan Spark 10M, https://www.tecan.com).

On each ELISA plate, we included orthopoxvirus-negative, orthopoxvirus-positive serum for internal quality control and for data normalization. In brief, we calculated the optical density difference between infected and noninfected cell lysate for each serum sample and then subtracted the orthopoxvirus-negative values from those of the orthopoxvirus-positive serum and test serum. The level cutoff was determined by the mean +4.65 SD for 24 blanks tested. By using this normalization, we considered any resulting value above the cutoff value as positive.

## Results

### Characterization and Isolation of CPXV

The presence of CPXV DNA in different samples was confirmed by PCR amplification using primers for the A27L orthopoxvirus gene and CPXV-specific genes D8L and D11L. PCR showed positive results for the cutaneous biopsy samples from postinfection days 29 and 31, fetus and placental samples on postinfection day 31, and vaginal swab samples from postinfection days 44 and 71 (Table). PCR amplification of plasma showed positive results on postinfection day 29 and negative results on postinfection days 11, 44, and 71 (Table).

In parallel, Vero cells inoculated with each sample showed the typical cytopathic effect resulting from orthopoxvirus infection after 5 days of incubation at 37°C. Cytopathic effects on Vero cells were observed for cutaneous biopsy samples from postinfection days 29 and 31 and for fetal and placental samples from postinfection day 31. Cytopathic effects were still observed from the vaginal swab sample from postinfection day 44. Results were negative for plasma at each day tested and for the vaginal swab sample from postinfection day 71 (Table). Quantification of CPXV-specific IgG in plasma samples on days −23, 11, 29, 44, and 71 detected CPXV-specific IgG in plasma samples from postinfection days 11 (data not shown) through 71.

### Serologic Results for the Patient’s Animals

For each of the patient’s animals, a veterinarian collected claw and plasma samples on August 24. Results of qPCR for CPXV DNA quantification were all negative. Serum samples analyzed by ELISA for CPXV-specific IgG detected no CPXV-specific IgG in serum from the rabbits and dogs but did detect CPXV-specific IgG in serum from 1 of the 3 cats (data not shown).

### Electron Microscopic Features of CPXV

**Figure 3:**
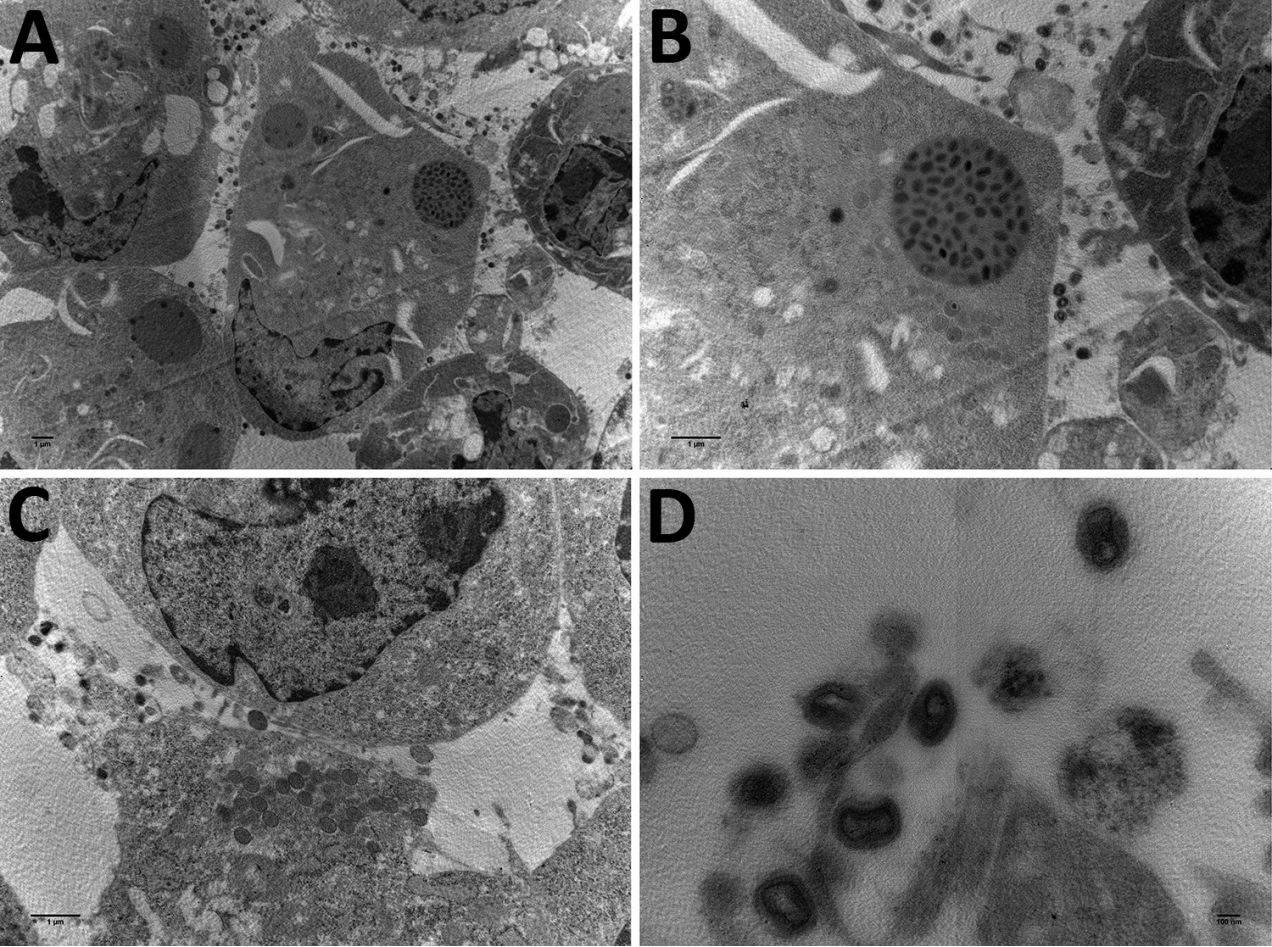
Electron microscopy images of cowpox virus CPXV-54-1716-France (CPXV-like 2), obtained from a pregnant woman in France, 2017. A) Ultrathin sections of BHK-21 cell at 42 hours after infection. Arrow indicates a typical inclusion in the cell cytoplasm. Original magnification ×4,600. B) Higher magnification of BHK-21 cell in panel A. Original magnification ×46,000. C) Ultrathin section of a BHK-21 cell with typical viral factories near the nucleus. Arrows indicate incomplete viruses. Original magnification ×10,500. D) Extracellular-enveloped viruses (arrow). Original magnification ×10,500.

When exploring the morphologic features of this CPXV during its viral replicative stage, we examined virus from fetal (Figure 3), placental, and vaginal swab samples. Electron microscopy showed a typical A-type inclusion in the cytoplasm (Figure 3, panels A, B), classifying this CPXV, named CPXV-54-1716-France, to the V+ subtype. We observed type B viral factories near the nucleus (Figure 3, panel C). We also observed extracellular enveloped viruses with a weight-shaped structure characteristic of CPXV, the nucleoside, containing genomic DNA and proteins involved in viral transcription (Figure 3, panel D).

### Phylogenetic Analysis

**Figure 4:**
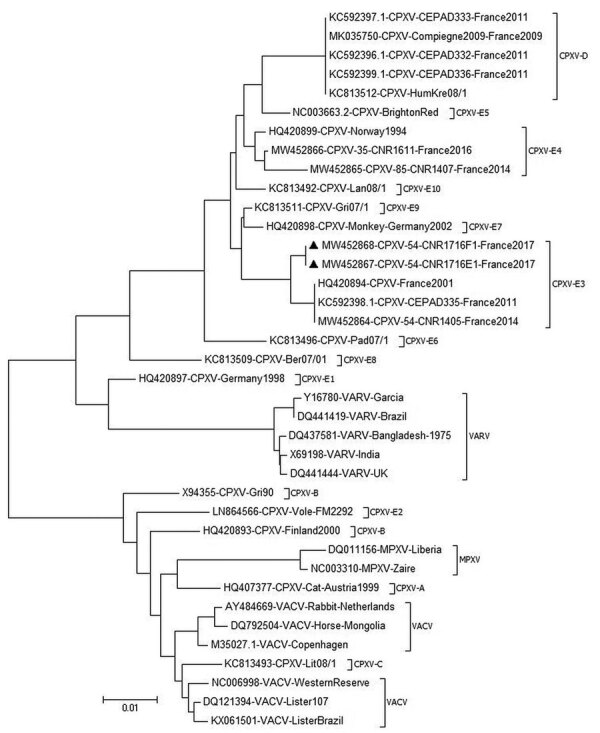
Phylogenic tree of CPXV collected from a woman in France (black triangles) and reference viruses. The tree was generated by using the maximum-likelihood method based on the nucleotide sequence of the A56R gene. The neighbor-joining algorithm was used to generate the initial tree. Evolutionary analyses were conducted in MEGA6 (https://www.megasoftware.net). GenBank accession numbers are provided. Scale bar indicates nucleotide substitutions/site. CPXV, cowpox virus; MPXV, monkeypox virus; VACV, vaccinia virus; VARV, varicella virus.

We performed phylogenetic analysis of the sample against the available A56R gene (Figure 4). We observed that the novel isolate, CPXV-54-1716-France, belongs to clade E3 (CPXV-like 2), for which the reference strain is the Nancy 2001 isolate (GenBank accession no. HQ420894.1).

## Discussion

CPXV represents a potential risk to human health, especially after the success of the worldwide smallpox vaccination campaign in 1979 and the subsequent cessation of vaccination. Smallpox vaccination is highly protective against other human-pathogenic orthopoxviruses, so stopping vaccination 40 years ago increased the probability of zoonotic orthopoxvirus infections in humans (*20*,*21*). In Europe, increasing numbers of documented infections with CPXV are being observed (*22*–*24*); lesions are usually localized to 1 extremity or body part (hands, face, neck, shoulders) (*25*). The case that we describe represents a rather atypical example of an orthopoxvirus infection of an orthopoxvirus-naive unvaccinated pregnant woman inducing fetal death. To date, the only mucous membrane–involved CPXV infections reported have been ocular (*5*,*26*,*27*), genital, or oral (*28*,*29*).

Historical studies have evaluated maternal outcomes in pregnancies complicated by smallpox. The overall case-fatality rate was estimated to be 34.3% (*30*). Because of the number of adverse events and the frequencies of complications after smallpox vaccination, maternal and fetal effects of smallpox vaccination during pregnancy were estimated. Despite the low incidence of fetal infection from the vaccinia virus (during smallpox vaccination) (*31*–*34*), some vaccinal complications, such as spontaneous abortion, congenital defects, stillbirth, preterm birth, or fetal vaccinia, have been described (*35*). Those studies have demonstrated that detection of infectious virus in fetal samples is consistent with the hypothesis that CXPVs are responsible for fetal death. The mechanisms governing the abortifacient activity of smallpox virus on gestating women has remained largely unexamined despite even acute maternal smallpox leading to spontaneous abortion, premature termination of pregnancy, and early postnatal infant death. 

For the patient we report, CPXV was found in high titers in fetal and placental samples only 17 days after infection. The virus was still detectable in vaginal samples at postinfection day 57. The presence of CPXV in fetal and placental samples supports the hypothesis that the CPXV infection was responsible for the fetal death.

In cases of congenital vaccinia, viremia in vaccinated pregnant women was suspected to follow direct infection of the fetus or indirect infection of the fetus by placental or amniotic membrane infection (*17*). Although viremia has been demonstrated for variola virus (*36*), human CPXV infections have not been correlated with viremia (*37*). For vaccinia virus, it seems to be a very rare event (*38*) linked to adverse effects of vaccination, for which viremia was common (*17*). We hypothesized that CPXV viremia probably appeared between postinfection days 11 to 29, explaining the CPXV dissemination to the fetus from the blood, because we demonstrated DNA in plasma samples from those days despite the lack of viremia. The presence of specific antibodies in plasma samples could be responsible for the lack of viremia detection. Moreover, the lack of viremia detection could result from the fact that our samples were plasma only, whereas viremia was detected in erythrocytes from whole blood in the case of variola virus (*37*). Despite the lack of viremia, systemic or even fatal infection may affect patients with an immune deficiency or site of atopy (*27*,*29*,*40*,*41*). Atopic dermatitis combined with pregnancy could probably account for an immunodeficiency state inducing the patient’s sensitivity to CPXV infection.

Rodents may serve as a natural reservoir for CPXV (*42*); however, transmission of CPXV to humans is mainly caused by direct contact with infected pet rats or cats. In this case, investigations of the patient’s domestic rabbits, cats, and dogs failed to demonstrate prevalence of CPXV infection among domestic pets. Because samples from pets arrive at the National Reference Center–Expert Laboratory for Orthopoxvirus months after the first samples from patients, it becomes difficult to retrace and investigate the route, initial host, or reservoir during the early stages of disease spread. The probable transmission of CPXV by 1 of the patient’s likely infected cats, for which CPXV-specific IgG was found, may have occurred without direct contact. Rather, it may have occurred through contact with a contaminated surface or object because of her atopic dermatitis and the ability of robust orthopoxviruses to survive for an extended time in the environment (*43*,*44*).

To complete the description of this new isolate, we explored its morphologic features in the viral replicative stage. Electron microscopy showed a typical A-type inclusion in the cytoplasm, classifying the CPXV-54-1716-France virus in the V+ subtype (*45*). For viruses within the genus *Orthopoxvirus*, the formation of A-type inclusion bodies is a characteristic of pathogenic infection.

Taking the A56R sequence comparison into consideration, we believe that CPXV-54-1716-France virus branched off from the proposed E3 subclade cluster in Europe (*46*). We also found in this cluster E3, CPXV isolated in 2011, 2014, and 2016, all circulating in the same region in France (*6*,*47*). This result strengthens the observations of an endemic circulating CPXV subclade E3 or CPXV-like 2 clade in France (*46*).

We also find in this cluster E3, CPXV isolated in 2011, 2014 and 2016, all circulating in the same french region

In conclusion, this case of fetal death highlights the risk for complications of orthopoxvirus infection during pregnancy. The correlates of progressive vaccinia and eczema vaccinatum (and now fetal vaccinia) that have been observed with cowpox (*48*) suggest that CPXV poses the same threats for infection complications as vaccinia virus.
